# Circadian rhythms of blood pressure in hypertensive patients with cerebral microbleeds

**DOI:** 10.1002/brb3.2530

**Published:** 2022-03-02

**Authors:** Yang‐Kun Chen, Wen‐Cong Liang, Shu‐Lan Yuan, Zhuo‐Xin Ni, Wei Li, Yong‐Lin Liu, Jian‐Feng Qu

**Affiliations:** ^1^ Department of Neurology Dongguan People's Hospital (Affiliated Dongguan Hospital Southern Medical University) Dongguan Guangdong Province China; ^2^ Department of Neurology Graduate School of Guangdong Medical University Zhanjiang Guangdong Province China

**Keywords:** blood pressure, cerebral microbleeds, cerebral small vessel disease, circadian rhythms

## Abstract

**Background:**

Whether the circadian rhythms of blood pressure (BP) contribute to the presence of cerebral microbleeds (CMBs) remains unknown. This study aimed to assess the relationship between nocturnal BP and CMBs in hypertensive patients.

**Methods:**

This prospective case‐control study recruited 51 hypertensive patients with CMBs and 51 hypertensive patients without CMBs, matched with age and gender, serving as controls. A 24‐h ambulatory BP monitoring was conducted in all subjects. Differences in ambulatory BP parameters between the two groups were compared. Logistic regression analyzes were conducted to investigate the relationship between the ambulatory BP parameters and presence of CMBs.

**Results:**

Patients with CMBs had a significant higher nocturnal mean SBP and lower relative nocturnal SBP dipping rate. Two logistic models were constructed to explore the association between ABPM indices and the presence of CMBs, adjusted with history of ischemic stroke and smoking. In model 1, higher nocturnal mean SBP positively correlated with presence of CMBs [standardized *β *= 0.254, odds ratio (OR) = 1.029, *p *= .041]. In model 2, the relative nocturnal SBP dipping rate was negatively correlated with CMBs (standardized *β *= −.363, OR = 0.918, *p *= .007). Only patients with deep CMBs had significant higher nocturnal mean SBP and lower relative nocturnal SBP dipping rate in comparison with those without CMBs.

**Conclusions:**

Higher nocturnal SBP and lower relative nocturnal SBP dipping rate may be associated with CMBs in hypertensive patients.

## Graphical abstract

In this study, we set out a case‐control study to assess the relationship between the circadian rhythms in short‐term BP and CMBs in patients with hypertension. We found that higher nocturnal mean SBP and lower relative nocturnal SBP dipping rate may be associated with a higher risk of presence of CMBs, which implicates abnormal circadian rhythms may play a role in the development of CMBs

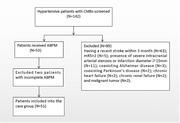

## 1 BACKGROUND

Cerebral microbleeds (CMBs), especially those located in deep brain structures and infratentorial regions, are one of the characteristics of cerebral small vessel disease (CSVD), together with lacunar stroke, white matter hyperintensities (WMHs) and enlarged perivascular spaces. Pathological study has confirmed that CMBs corresponded to the deposition of hemosiderin‐containing macrophages around the tiny blood vessels where leakage or rupture occurs, which in turn impairs the brain parenchyma (Wardlaw et al., 2013). Studies have shown that CMBs can predict the recurrence of ischemic and hemorrhagic strokes (Boulanger et al., 2006; Charidimou et al., 2013; Fan et al., 2003; Jeon et al., 2007), indicating the potential clinical significance of CMBs.

CMBs occur in 16.1%–73% of hypertensive individuals (Henskens et al., 2008; Koennecke, 2006; Lyu et al., 2020), and it is independently related to blood pressure level (Cordonnier et al., 2007; Lyu et al., 2020; Romero et al., 2014) and blood pressure variability (BPV) (Liu et al., 2012). In addition to average BP levels, the circadian rhythm in BP, an inherent characteristic of BP, has been of increasing concern by researchers in recent decades. It is an important parameter of how the cardiovascular autonomic system modulates the BP. Actually, abnormal circadian rhythms of BP, for example, reversal‐dipping of nocturnal BP, elevated the risk of cardiovascular events, or stroke. BP indices obtained by Ambulatory BP monitoring (ABPM) have been shown more closely to hypertension‐related organ impairment than single office measurement (Dolan et al., 2005).

However, data on the circadian rhythm of BP assessed by ABPM and CMBs are limited. Physicians may be more concerned why some hypertensive patients present with CMBs, whereas others do not. Therefore, we set out this case‐control study to assess the relationship between the circadian rhythms in short‐term BP and CMBs in patients with hypertension. We hypothesized that the presence of CMBs may be more common in hypertensive patients with higher nocturnal BP or inadequate dipping of nocturnal BP.

## 2 METHODS

### 2.1 Study participants

This was a prospective case‐control study conducted in the Department of Neurology, Dongguan People between September 1, 2017 and July 30, 2019. Hospitalized patients with hypertension (as defined below) and MRI (including SWI sequence) conducted were screened. Fifty‐one hypertensive hospitalized Chinese Han patients with CMBs were included, serving as the case group. These patients were admitted in hospital due to dizziness (*N* = 17), vertigo (*N* = 14), gait disturbance (*N* = 10), cognitive decline (*N* = 7), and headache (*N* = 3). The inclusion criteria were: (1) age between 40 and 85 years; (2) a diagnosis of hypettention. Hypertension defined as either SBP ≥140 mmHg, or DBP ≥90 mmHg at three different timepoints using a mercury sphygmomanometer. Patients with documented history of hypertension were also judged to have hypertentison; (3) Modified Rankin scale (mRS) ≤ 2; and (4) presence of CMBs. CMBs were defined as small and round lesions (diameter 2−10 mm) with signal loss on SWI sequence in MRI, excluding cavernous malformation, basal ganglia calcification, and flow void of the pial blood vessels. Exclusion criteria were: (1) a stroke within 3 months; (2) moderate‐to‐severe intracranial arterial stenosis or any infarct with a diameter > 15 mm; (3) had other central nervous system diseases other than stroke, for example, brain tumor, Parkinson's disease, Alzheimer's disease; (4) moderate‐to‐severe intracranial arterial stenosis or any infarct with a diameter > 15 mm; (5) had other centrprimary hyperaldosteronism, pheochromocytoma or hypercortisone; and (6) presence of contraindication of MRI examination, for example, cardiac pacemaker, ferromagnetic aneurysm clip, or claustrophobia. Patients without CMBs might serve as controls group and they matched with the list of the patients in the case group during or close to the same period in terms of age (± 3 years) and gender. The same inclusion and exclusion criteria were used except for the presence of CMBs. This study was approved by the Ethics Committee of Dongguan People in accordance with Declaration of Helsinki. All subjects provided their written informed consent. This study was registered in www.chictr.org.cn (No. ChiCTR1800016760).

### 2.2 Collection of clinical and laboratory data

Information on participants’ demographic data (sex and age), vascular risk factors (hypertension, diabetes mellitus, hyperlipidemia and smoking), history of stroke, and treatment. Information was collected from their medical records.

### 2.3 Twenty‐four‐hour ambulatory BP monitoring (24‐h ABPM)

Twenty‐four‐hour ABPM was performed in all participants after MRI examination during their hospital stay using an automated device (DMS‐ABP, DM software Inc, California, American). The median intervals between MRI examination and ABPM was 3 (interquartile range 2–4) days. Participants underwent ABPM were requested to avoid physical exercise and excessive movement on their nondominant arm during measurements. Readings were performed every 30 min during daytime (08:00 am to 22:59 pm) and every 60 min during the nighttime (23:00 pm to 07:59 am). Automated calculated parameters of ABPM were adopted as follows: (1) mean SBP and DBP 24 h, daytime, and nighttime; (2) the weighted standard deviation (SD) of SBP and DBP across 24 h, defined as the mean of day and night SD values corrected for the number of hours included in each of these periods; and (3) the coefficient of variation (CV) defined as the ratio between SD and the mean SBP or DBP at the same periods. Additionally, we calculated the circadian variation using relative nocturnal systolic dip as follows: ([daytime SBP‐nighttime SBP]/daytime SBP) × 100.

### 2.4 MRI measurement

A 3.0 T system (Skyra, Siemens Medical, Germany) MRI examination was performed for each participant within 72 h of admission, including T1‐weighted imaging (T1WI), T2‐weighted imaging (T2WI), fluid‐attenuated inversion recovery (FLAIR), and SWI. The parameters of MRI examination referred to our previous study (Chen et al., 2021). The MRI parameters for SWI were TR/TE/excitation = 27/20/1, FOV = 220 mm, slice thickness/gap = 3 mm/0.6 mm, matrix 256 × 256, and time of acquisition = 2 min 28 s.

A trained and experienced neurologist (YKC) who was blinded to clinical data measured the MRI variables as follows: (1) lacunar infarcts (lacunar infarcts were defined as infarcts with the greatest diameter less than 15 mm on MRI, with hypointensities on T1WI and hyperintensities on T2WI and FLAIR. The number of lacunar infarcts was counted); (2) WMHs [the severity of WMHs was graded using the 4‐point Fazekas’ scale (Fazekas et al., 1987) with periventricular hyperintensities (PVH) and deep white matter hyperintensities (DWMH) scored on FLAIR, respectively]; and (3) CMBs [CMBs were defined as small and round lesions (diameter 2−10 mm) with signal loss on SWI‐MRI, excluding cavernous malformation, basal ganglia calcification, and flow void of the pial blood vessels (Greenberg et al., 2009)]. The location of CMBs was divided into strict lobar, deep (with and without lobar and infratentorial region), and isolated infratentorial regions.

Numbers of CMBs was counted. The intrarater reliability of the MRI measurements were good: kappa value was .79 and .76 for CMBs and WMHs; intraclass correlation coefficient (ICC) was .82 for number of lacunar infarcts.

### 2.5 Statistically analysis

Statistical analyses were performed using statistical software (SPSS for Windows v.19.0; SPSS Inc., Chicago, IL, USA). Clinical and ABPM parameters were compared between patients with and without CMBs using *χ* ^2^ tests (frequency), two independent‐samples *t*‐tests (normally‐distributed variables), and Mann–Whitney *U* tests (distorted variables). Variables with *p *< .1 in univariate comparisons, as well as age, were included into subsequent multivariable logistic regressions. Normality of variable were tested using one‐sample Kolmogorov–Smirnov test. Multivariable logistic regressions analyses with the stepwise method were conducted to examine the independent contributing factors for CMBs. The significance level was set at *p* < .05 (two‐sided). One‐way analysis of variance (ANOVA) was conducted to compare the differences of BP indices among four groups in terms of no CMBs, strict lobar, deep and isolated infratentorial CMBs with least‐significant difference (LSD) method.

## 3 RESULTS

**FIGURE 1:**
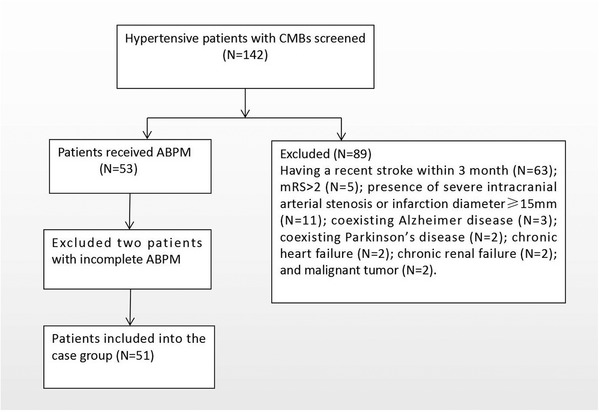
Flow chart of the recruitment of case group

During the study period, 212 hypertensive patients with CMBs were screened. The recruitment of the study participants were shown in Figure 1. A final total of 51 patients with CMBs were recruited, while 51 gender‐ and age‐matched hypertensive patients without CMBs were included in the control group.

The comparisons of clinical variables were shown in Table 1. Patients with CMBs had more previous ischemic stroke and had a trend of higher frequency of smoking and antiplatelet agents.

**TABLE 1:** Comparisons of clinical variables between hypertensive patients with and without CMBs

	With CMBs	Without CMBs		
Variables	*n* = 51	*n* = 51	*T*/*z*/*χ* ^2^	*p*
Clinical variables				
Age (years)	68.5±8.7	68.4±8.4	−0.081	.935
Sex (men), *n* (%)	26 (51.0%)	26 (51.0%)	0.000	1.000
Diabetes mellitus	10 (19.6%)	13 (25.5%)	0.505	.477
Hyperlipidemia	20 (39.2%)	19 (37.3%)	0.042	.839
Smoking	15 (29.4%)	7 (14%)	3.520	.061
Ischemic stroke	15 (29.4%)	4 (7.8%)	7.826	.010
Treatment (*n*%)				
Antihypertensive agents	24 (47.1%)	20 (39.2%)	0.639	.424
Antiplatelet agents	11 (21.6%)	4 (7.8%)	3.830	.091
Glucose‐lowering agents	7 (13.7%)	5 (9.8%)	0.378	.539
Satins	7 (13.7%)	6 (11.8%)	0.088	.767
MRI features				
PVH	3 (1–3)	0 (0–1)	−4.956	<.001
DWMH	2 (1–3)	0 (0–0)	−6.310	<.001
No. of lacunar infarcts	1 (0–3)	0 (0–0)	−4.273	<.001

CMBs = cerebral microbleeds; DWMH = deep white matter hyperintensities; PVH = periventricular hyperintensities; CMBs = cerebral microbleeds.

Of the 51 patients with CMBs, 10 (19.6%) had strict lobar CMBs, 32 (62.7%) had deep CMBs (with/without lobar or infratentorial CMBs), 9 (17.6%) had isolated infratentorial CMBs.

### 3.1 ABPM indices and presence of CMBs

Twenty‐four‐hour mean SBP and DBP, daytime mean SBP and DBP, CV of SBP and DBP did not differ significantly between patients with and without CMBs. Patients with CMBs had a significant higher nocturnal mean SBP and lower relative nocturnal SBP dipping rate (Table 2). Among all the subjects of both groups, only one patient without CMBs was extreme nocturnal SBP dipper (≥20% dipping at nighttime). Two logistic models were constructed to explore the association between ABPM indices and the presence of CMBs. As nocturnal mean SBP and relative relative nocturnal SBP dipping rate were highly correlated with each other, they were separately entered into the logistic regression models, together with ischemic stroke, smoking and use of antihypertensive agents (*p *< .1 in univariate analysis). In model 1, higher nocturnal mean SBP [standardized *β *= .254, odds ratio (OR) = 1.029, *p *= .041], and ischemic stroke (standardized *β *= .312, OR = 4.255, *p *= .020) positively correlated with presence of CMBs. In model 2, the relative nocturnal SBP dipping rate (standardized *β *= −.363, OR = 0.918, *p *= .007) was negatively correlated with CMBs, after adjusted by the other variables (Table 3).

**TABLE 2:** Comparisons of blood pressure variability between patients with and without CMBs

	With CMBs	Without CMBs		
Variables	*N* = 51	*N* = 51	*T*/*z*/*χ* ^2^	*p*
24‐h mean SBP (mmHg)	138.5±13.7	133.7±14.1	−1.542	.126
24‐h mean DBP (mmHg)	77.9±9.4	76.1±11.3	−0.910	.365
CV of 24‐h SBP (%)	10.6±2.6	10.7±2.9	0.153	.879
CV of 24‐h DBP (%)	14.4±4.5	14.7±3.9	0.369	.713
Daytime mean SBP (mmHg)	139.1±13.4	136.8±14.5	−0.853	.396
Daytime mean DBP (mmHg)	78.4±9.7	77.1±12.2	−0.584	.561
CV of daytime SBP (%)	10.2±2.6	9.9±2.3	−0.713	.477
CV of daytime DBP (%)	14.2±5.1	13.5±4.0	−0.753	.453
Nocturnal mean SBP (mmHg)	138.1±15.6	130.0±16.4	−2.530	.013
Nocturnal mean DBP (mmHg)	76.8±9.2	72.8±11.0	−1.967	.052
CV of nocturnal SBP (%)	9.5±3.6	10.6±2.8	1.662	.100
CV of nocturnal DBP (%)	13.5±5.4	14.7±5.6	1.067	.289
Relative nocturnal SBP dipping rate (%)	0.7±6.8	4.9±8.0	2.817	.006
Nocturnal DBP fall rate (%)	1.8±7.4	4.7±9.2	1.758	.082

CMBs = cerebral microbleeds; SBP = systolic blood pressure; DBP = diastolic blood pressure; CV = coefficient of variance.

**TABLE 3:** Multivariable logistic regressions models of presence of CMBs in hypertensive patients

Variables	Std. *β*	OR	95% CI	*p*	*R* ^2^ change
*Model 1*					
History of ischemic stroke	.312	4.255	1.258–14.393	.020	.104
Nocturnal mean SBP	0.254	1.029	1.001–1.057	.041	.159
Smoking	0.206	2.464	0.860–7.057	.093	.192
Antiplatelet agents	0.082	1.521	0.321–7.204	.597	.195
Age	0.023	1.006	0.957–1.057	.825	.196
*Model 2*					
History of ischemic stroke	0.358	5.272	1.535–18.103	.008	.101
Relative nocturnal SBP dipping rate	−0.363	0.918	0.862–0.977	.007	.199
Smoking	0.229	2.715	0.916–8.046	.072	.237
Antiplatelet agents	0.119	1.831	0.394–8.517	.440	.243
Age	−0.005	0.989	0.939–1.042	.677	.245

CMBs = cerebral microbleeds; SBP = systolic blood pressure.

### 3.2 Location of CMBs and nocturnal SBP

**FIGURE 2:**
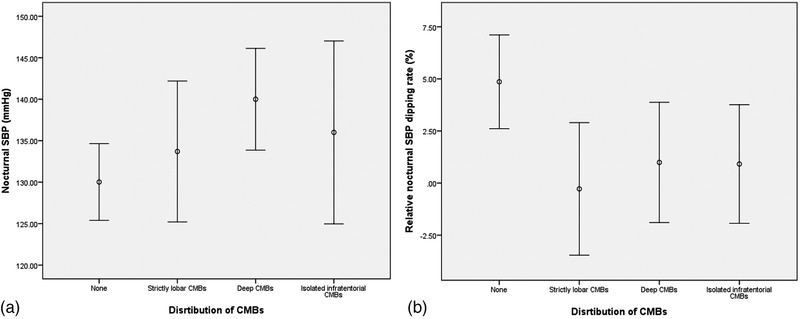
Nocturnal SBP, relative nocturnal SBP dipping rate, and location of CMBs. (a) Nocturnal mead SBP. Deep CMBs vs. none, *p *= .007. (b) Relative nocturnal SBM dipping rate

One‐way ANOVA analysis compared the nocturnal mean SBP and relative nocturnal SBP dipping rate among patients without CMBs, with strict lobar CMBs, deep CMBs, and isolated infratentorial CMBs, corrected with the LSD method. Compared with patients without CMBs, only patients with deep CMBs had significant higher nocturnal mean SBP (140 ± 17 mmHg vs. 130.3 ± 16.1 mmHg, corrected *p *= .009), as well as lower relative nocturnal SBP dipping rate (1.0% ± 8.0% vs. 4.7% ± 8.0%, corrected *p *= .033), whereas those with strict lobar CMBs or isolated infratentorial CMBs did not. The three groups did not differ significantly in terms of nocturnal mean SBP and relative nocturnal SBP dipping rate (Figure 2).

## 4 DISCUSSION

The present study demonstrated that higher nocturnal mean SBP and lower relative nocturnal SBP dipping rate were associated with a higher risk of presence of CMBs, implicating abnormal circadian rhythms may play a role in the development of CMBs. Furthermore, subjects with deep CMBs seemed to have higher nocturnal mean SBP and lower relative nocturnal SBP dipping rate, in comparison with those without CMBs.

A few studies have demonstrated that both daytime SBP and nocturnal SBP are associated with the development of CMBs in hypertensive patients (Henskens et al., 2008; Lyu et al., 2020). In other studies (Klarenbeek et al., 2013; Staals et al., 2009), researchers also found a clear association of both higher day and night BP levels with CMB incidence in lacunar stroke patients. Besides, an excessive morning surge in SBP was independently associated with a higher risk of development of CMBs (Zhang et al., 2019). Most of these studies recruited subjects with acute ischemic stroke. However, rhythm of ABPM may change in the acute phase of ischemic stroke. In order to avoid confounding effect, we chose hypertensive participants who were medically stable and did not recruit those with acute ischemic stroke. In line with the above studies, we also found that higher nocturnal mean SBP was significantly associated with CMBs in an age‐and gender‐balanced hypertensive case control sample. The present study indicated that lower relative nocturnal SBP dipping rate was also predictive of CMBs, similar to Kwon and colleagues’ study (Kwon et al., 2014), which reported that CMBs are independently associated with reverse dipping on ABPM in hypertensive stroke patients after adjustment for age, gender, and cardiovascular risk factors. Of the three types of distribution of CMBs, deep CMBs, which may represent hypertensive CSVD‐related CMBs, correlated with these two nocturnal SBP‐related indices most significantly, which was rarely reported in previous studies. It might be partly attributed to the fact that the number of cases with strict lobar CMBs and isolated infratentorial CMBs were small. Furthermore, we could not preclude the coexistence of CAA pathologies in these patients with deep CMBs as some of them might also have lobar CMBs (Pasi et al., 2018). Our study failed to confirm an apparent association between CMBs and daytime SBP. The reason for these inconsistencies might be that our study sample (e.g., over 40% subjects on hypertensive treatment) differed from the previous studies. In other words, in the case of similar daytime SBP, patients with CMBs were more likely to have higher nocturnal mean SBP as well as lower relative nocturnal SBP dipping rate.

The mechanism behind abnormal circadian rhythm‐related CMBs remains less confirmative. First, hypertension is associated with a higher risk of the development of CMBs. As hypertension develops, the small arteries and capillaries are exposed to a high level of pressure, which damage the integrity of vascular endothelium, blood–brain barrier (BBB) impairment with subsequent extravasation of blood. Second, reduced nocturnal dipping is associated with the degree of target organ damage in hypertensive patients (Parati & Staessen, 2007). BBB is easy to be disrupted by BP‐related hemodynamic factors, for example, reversal‐dipping of nocturnal BP. Moreover, there is a lot of evidence that abnormal circadian rhythms damages CSVD. For example, it was reported in literature that alteration of blood pressure induced by sympathetic neural activation could cause higher night BP and reversal‐dipping of nocturnal BP in patients with moderate‐to‐severe OSA (Song et al., 2017). This alteration in blood pressure could cause shearing forces to the cerebral small vessel as well as injure arterioles (Filomena et al., 2015), which lead to CSVD. Besides, moderate‐to‐severe OSA may be associated with the hemorrhagic change (mainly CMBs) indicators of CSVD (Song et al., 2017). Therefore, it may be suggested that abnormal circadian rhythms of BP is an important factor occurring in OSA could cause CSVD. Furthermore, circadian BP changes are conditioned by nocturnal hormonal changes that include autonomic nervous system. It may be hypothesized that abnormal fluctuations in levels of hormones are responsible for higher nighttime BP.

Data on the relationship between CV or SD of BP and CMBs is very limited. Liu et al. (2012) suggested that CMBs are associated with CV in BP with in patients with a history of ischemic stroke within 1 to 6 months. However, Sabayan et al. (2013) did not clarify a significant association between SD of BP and CMBs, using visit‐to‐visit BP measurement. However, we did not find a significant association between the CV and the presence of CMBs. BPV or SD in Liu et al. and Sabayan et al.’s study was measured using long‐term visit‐to‐visit BP rather than ABPM as in the present study. The effects of BPV indices on CMBs need further assessment.

In the univariate comparisons, CMBs group had more ischemic stroke, smokers, and antiplatelet users. In logistic regression analysis, history of ischemic stroke was still significantly associated with the presence of CMBs after adjusted by BP indices, while smoking and receiving antiplatelet agents was not. Additionally, patients with and without CMBs had a comparable frequency of antihypertensive agents (47.1% vs. 39.2%, *p *= .242) and other comorbidities. We did not exclude those receiving antihypertensive agents, which may affect their baseline BP level, but the two groups did not differ in terms of frequency of antihypertensive agents. Thus, whether receiving antihypertensive agents or not might not affect the result significantly.

In the present study, only around 40% of the hypertensive patients were receiving antihypertensive agents. According to a national epidemiologic survey (Mahajan et al., 2020) in elderly in China from 2012 to 2015, the treatment and control rate of hypertension is only 51.4% and 18.2%, respectively. The fact that the medication rate in our participants was a little lower than it was in the community cohort may be due to the limited representativeness of the study subjects.

The strength of our study is that we collected a gender‐ and age‐matched patient group with hypertension, as hypertension and age are considered as two major risk factors of CMBs. For physicians, they may concern which hypertensive patients have higher risk of CMBs. Our study may add to the evidence of association between CMBs and ABPM, as such data are still limited. Furthermore, we conducted an analysis on the location of CMBs and ABPM, which was rare in previous studies. However, our study also had several limitations. First, the assessment of MRI and ABPM was performed cross‐sectionally, leading not able to demonstrate the causality. Second, ABPM was conducted once during hospitalization instead of home environment. They might not have the same BP circadian rhythm as that at home. It may lead to limited generalization of the finding in this study. Third, we did not exclude those with ischemic stroke, which may affect the homogeneity of the study sample. But we have ruled out those with large artery disease, larger infarcts and recent infarction. Thus, the finding in this study is restricted to Chinese Han hypertensive patients, and is not applicable to those with a recent stroke or previous stroke due to larger artery disease. Finally, the sample size was small. Patients with strict lobar CMBs had only 8 and that is 11 in the group with isolated infratentorial CMBs. It is not enough to explore ABPM parameters and the different locations of CMBs in more detail.

In conclusion, our results show that higher nocturnal mean SBP and lower relative nocturnal SBP dipping rate may be associated with a higher risk of presence of CMBs, which implicate abnormal circadian rhythms may play a role in the development of CMBs.

## ETHICS STATEMENT

This study was approved by the Ethics Committee of Dongguan People in accordance with Declaration of Helsinki.

## AUTHOR CONTRIBUTIONS

YKC conceived and designed the study. WL, ZXN, WCL, and SLY recruited the subjects. YKC conducted the MRI assessment. YKC and SLY wrote the paper. YLL and JFQ reviewed and edited the manuscript. All authors read and approved the manuscript.

### PEER REVIEW

The peer review history for this article is available at https://publons.com/publon/10.1002/brb3.2530

## Data Availability

The data used to support the findings of this study are available from the corresponding author upon request.
